# Self-Perceptions of Aging, Cognitive Function, and Physical Activity

**DOI:** 10.1093/geroni/igaa057.3261

**Published:** 2020-12-16

**Authors:** Caitlin Connelly

**Affiliations:** University of Massachusetts Boston, Boston, Massachusetts, United States

## Abstract

Stereotype embodiment theory suggests that internalized aging stereotypes will influence subsequent physical and cognitive health for older adults. This is proposed to occur through behavioral, physiological, and psychological pathways. Guided by stereotype embodiment theory, this study examined the how self-perceptions of aging are associated with cognitive function and the mediating role of physical activity as a behavioral pathway. The sample consists of 7,990 community-dwelling older adults age 65 from the Health and Retirement Study. Cross-sectional data analyses were conducted using bivariate and multivariate linear regression. Positive self-perceptions of aging were significantly associated with better cognitive function. Physical activity partially mediated the association between self-perceptions of aging and cognitive function. Findings suggest that self-perceptions of aging are important for cognitive function and physical activity may help to explain this relationship. Self-perceptions of aging may serve a possible intervention point to increase physical activity engagement and improve cognitive function.

